# Approach and Clinical Practice of Functional Movement Disorders Among Neurologists in Saudi Arabia

**DOI:** 10.7759/cureus.32770

**Published:** 2022-12-21

**Authors:** Maryam Alqassas, Mohammad H Alatiyah, Sarah S Aldharman, Mohammed Z Alburayman, Mohammed H Alrashed, Abdulmuhsin A Al-Sultan, Reham Alrahil

**Affiliations:** 1 Department of Medicine, Ministry of National Guard Health Affairs, Jeddah, SAU; 2 College of Medicine, King Faisal University, Al-Ahsa, SAU; 3 College of Medicine, King Saud Bin Abdulaziz University for Health Sciences, Riyadh, SAU; 4 College of Medicine, University of Tabuk, Tabuk, SAU

**Keywords:** practice, approach, saudi arabia, neurologists, functional movement disorders

## Abstract

Background

Functional movement disorders (FMDs) are neurological disorders that consist of abnormal and involuntary movements that have no specific organic cause. Given the prevalence of FMDs and the scarcity of information on neurologists' approaches to FMDs, we aimed to assess the neurologists' approaches and clinical practice in managing FMDs in Saudi Arabia.

Methods

A validated online questionnaire in English was used. The data were collected through a self-reported questionnaire. The data were analyzed using the statistical software IBM SPSS version 22 (SPSS, Inc., Armonk, NY).

Results

A total of 231 neurologists completed the study survey. A total of 129 (55.8%) were males. Regarding the predictors for a diagnosis other than FMD, the highest rating predictor was for evidence of physical injury and lack of psychiatric history of psychological stressors, while the lowest rating was for the male gender. Regarding the effective treatment strategies of FMD, the most effective treatment strategy reported by the clinicians was patient education, while the least was alternative or complementary medicine. The management ability of clinicians was generally restricted by cultural beliefs about psychological illnesses and the availability of referral services. The reported predictors by clinicians for a better prognosis of FMD include acceptance of the diagnosis by the patient followed by identification and management of psychological stressors and concurrent psychiatric disorder and a supportive social network. Generally, the most used terminology in this study was "functional movement disorder".

Conclusions

There is a variation in the approach and clinical practice of FMD among neurologists in Saudi Arabia. Shared knowledge regarding diagnosis and effective management is crucial. Collaborative efforts are required to establish practice guidelines in the future.

## Introduction

Functional movement disorders (FMDs) are neurological disorders that consist of abnormal and involuntary movements that have no specific organic cause [1]. They are sometimes referred to as conversion disorders and psychogenic movement disorders (PMD) [1]. The Diagnostic and Statistical Manual of Mental Disorders (DSM-5) provides certain criteria for conversion disorder, defined to consist of a functional neurological symptom that is incompatible with recognized neurological or medical conditions. Incompatibility is measured during clinical examination and bedside tests that are specific to FMDs [2,3].

Although no specific organic cause has been attributed to FMDs, multiple etiologies may explain its pathogenesis, including biological factors, psychological stressors, and social issues [4]. A study showed that among 550 new patients referred for assessment at tertiary care movement disorders facilities, 45 (8.2%) were diagnosed with FMD, compared to the previous year, when 665 new patients were assessed and 5.1% were diagnosed with FMD [5]. This reflects a 60.1% rise during the COVID-19 pandemic which may be attributed to the increased psychological and other stressors during the COVID-19 pandemic [5]. Many studies have shown a high tendency to develop anxiety and depression in patients with FMDs [6]. They are often difficult and time-consuming to diagnose and manage, seen frequently in neurology clinics, and many neurologists face difficulty in taking care of such patients. Therefore, patients are often misdiagnosed and inappropriately managed to lead to costly, inappropriate treatment, and poor prognosis [7].

A thorough history and clinical examination may give clues to FMDs however none are very specific, and neurologists thus need to be trained to look at the whole picture. There has been a significant advancement in this area. Our improved understanding of the pathophysiology is consistent with the "classical view" that the condition is caused by an emotional disturbance [4]. A study was conducted in China to assess neurologists’ knowledge and clinical practice, both of which showed interesting approaches to FMDs and illustrate substantial gaps are there in access to treatment and the education of FMD diagnosis and vary among Chinese neurologists, also differed from international peers respectively [8,9]. Given the frequency of FMDs, the suffering of its patients, the high likelihood of its mismanagement, and more importantly the limited information on neurologists’ approach to FMDs, especially in the Middle East region, we aimed to assess the neurologists’ approaches and clinical practice in managing FMD in Saudi Arabia.

## Materials and methods

A cross-sectional questionnaire-based study was carried out in Saudi Arabia between August 2022 and November 2022. The target population was the neurologists who may be exposed to FMD from different areas of Saudi Arabia (Central, Southern, Eastern, Western, and Northern). The data were collected by a self-administered questionnaire. The questionnaire was distributed electronically via Google Forms. The data were incorporated into Microsoft Excel and then analyzed employing the Statistical Product and Service Solutions (SPSS) (IBM SPSS Statistics for Windows, Version 22.0, Armonk, NY).

Inclusion criteria and exclusion criteria

The neurologists who may be exposed to FMD working in Saudi Arabia were included in this study. Participants who did not fill out the whole questionnaire or did not agree to participate were excluded from the study.

Sampling technique and sample size calculation

The OpenEpi® version 3.0 (The OpenEpi Project, Atlanta, Georgia) software was employed to calculate the sample size. The representative sample size required was 169, with a margin error of 5%, a confidence level of 95%, and a population size of 300 [10]. We planned to get more than the calculated sample size to conquer any anticipated exclusions. Non-probability consecutive sampling technique was used.

Data collection instruments and procedures

A validated online questionnaire in English was used. The data were collected through a self-report questionnaire. A 21-item questionnaire adapted from the recent Modified Depression Scale (MDS) version [9]. The questionnaire has four sections: the first section includes questions about the diagnosis of FMD. The second section covers treatment. The third component dealt with prognosis. The fourth section includes questions about terminology related to FMD.

An electronic Google Form survey was distributed online. All information was kept secretive and utilized only used for scientific research, and participation in this study was voluntary with the participants' informed consent obtained on the first page prior to filling out the questionnaire. Before beginning the study, ethical approval was acquired. Ethical approval was obtained from the research ethics committee at King Faisal University before initiating the study (reference No. KFU-REC-2022-MAY-ETHICS9).

Data management and statistical analysis

After distributing the questionnaire, they were checked for completeness and any missing information. Then, the data were entered into Microsoft Excel. After data were extracted, it was revised, coded, and fed to the statistical software IBM SPSS. All statistical analysis was done using two-tailed tests. P value <0.05 was statistically significant. Descriptive analysis based on frequency and percent distribution was done for all categorical variables while the mean rate scale for different opinion items was calculated for ranking. Also, Spearman correlation analysis was performed within rating questions. Fellowship training and years of practice in MDs influence the number of FMD patients assessed per month was assessed using the Pearson chi-square test. Rating of opinion items, terms use, and all other factors were graphed according to the frequency of answers category and overall mean rank.

## Results

A total of 231 respondents completed the study survey. Respondents' ages ranged from 25 to 65 years with a mean age of 26.8 ± 13.9 years old. A total of 129 (55.8%) were males and 163 (70.6%) were resident doctors, 32 (13.9%) were associate chief physicians, and 22 (9.5%) were chief physicians. Exact 158 (68.4%) had no fellowship training in movement disorders, 121 (52.4%) had years of experience less than five years, and 72 (31.2%) had six to 10 years of experience. A total of 169 (73.2%) were in the neurology department, and 47 (20.3%) were physicians. As for the number of patients with PMD that you see per month, 85 (36.8%) reported for less than one patient, 59 (25.5%) reported for one to three patients while 37 (16.0%) see four to six patients, and 12.6% were uncertain. Considering the number of patients with all movement disorders respondents see per month, 133 (57.6%) reported or less than 30, 36 (15.6%) see 31-45 patients, seven (3.0%) see more than 80 patients, and 12.6 were uncertain. The demographics and the overall practice of respondents are shown in Table 1.

**Table 1:** Demographics and the overall practice of the participants PMD: Psychogenic movement disorders

Variable	No	%
Age in years		
25-35	189	81.8%
36-45	31	13.4%
46-55	8	3.5%
56-65	3	1.3%
Gender		
Male	129	55.8%
Female	102	44.2%
Professional title		
Resident doctor	163	70.6%
Visiting staff	14	6.1%
Associate chief physician	32	13.9%
Chief physician	22	9.5%
Length of fellowship training in movement disorders		
None	158	68.4%
One year	24	10.4%
Two years	22	9.5%
Three years	12	5.2%
Four years	15	6.5%
Years in practice		
<5 years	121	52.4%
6-10 years	72	31.2%
11-15 years	19	8.2%
16-20 years	11	4.8%
>21 years	8	3.5%
Department		
Neurology	169	73.2%
Specialty of movement disorder	2	.9%
Physician	47	20.3%
Psychiatry or Psychology	7	3.0%
Others	6	2.6%
Which best describes the number of patients with PMD that you see per month?		
<1	85	36.8%
1-3	59	25.5%
4-6	37	16.0%
7-10	12	5.2%
>11	9	3.9%
Uncertain	29	12.6%
Which best describes the number of patients with all movement disorders you see per month?		
<30	133	57.6%
31-45	36	15.6%
46-60	11	4.8%
61-80	15	6.5%
>80	7	3.0%
Uncertain	29	12.6%

Table 2 shows the relation between fellowship training and years of practice, and the number of FMD patients assessed per month. The number of FMD patients correlated with the number of patients with all movement disorders seen in the clinic monthly (r = 0.247, P < 0.001). Also, 47.9% of respondents with the length of fellowship training in movement disorders see more than three FMD patients per month versus 32.9% of others with no fellowship with recorded statistical significance (P = .028). Additionally, 43.6% of respondents with practice years in the MDs subspecialty of six years or more see more than three patients per month compared to 32.2% of others with fewer practice years (P = .049).

**Table 2:** Fellowship training and years of practice in MDs influence the number of FMD patients assessed per month * P < 0.05 (significant) MDs: Movement Disorders FMD: Functional movement disorders

	Number of patients with FMD that you see per month				p-value
	< 3 patients (n = 144)		>3 patients (n = 87)		
	No	%	No	%	
Length of fellowship training in movement disorders					.028*
None	106	67.1%	52	32.9%	
1-3 or longer	38	52.1%	35	47.9%	
Years of practice in MDs subspecialty					.049*
<5 years	82	67.8%	39	32.2%	
6-21 years or longer	62	56.4%	48	43.6%	

**Figure 1:**
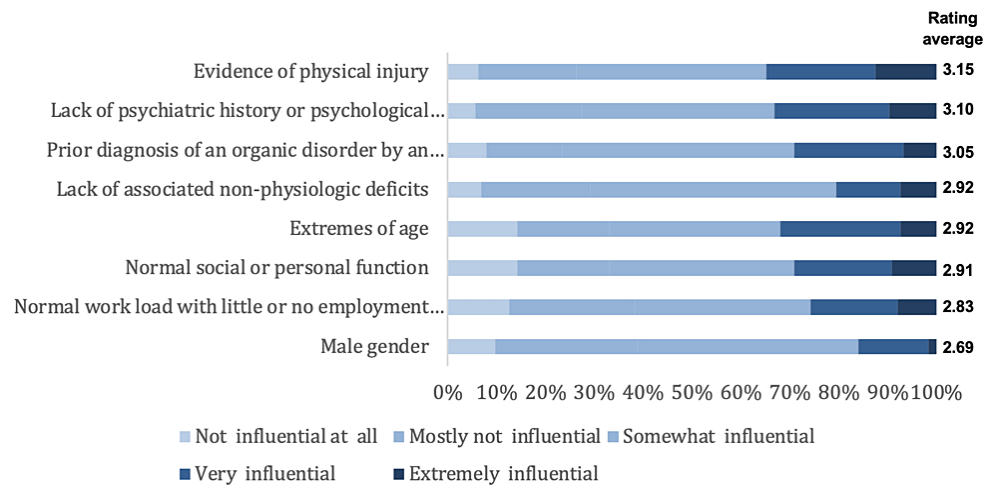
Influence of predictors for the non-FMD diagnosis FMD: Functional movement disorders

**Figure 2:**
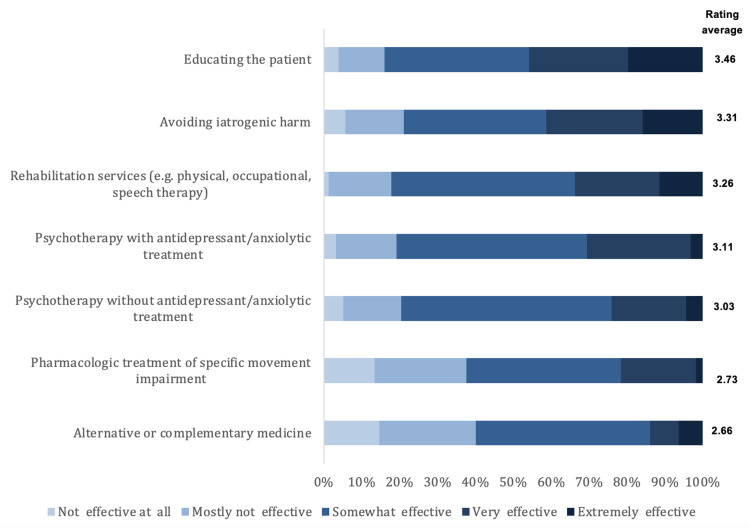
The effectiveness of treatment strategies for FMD FMD: Functional movement disorders

**Figure 3:**
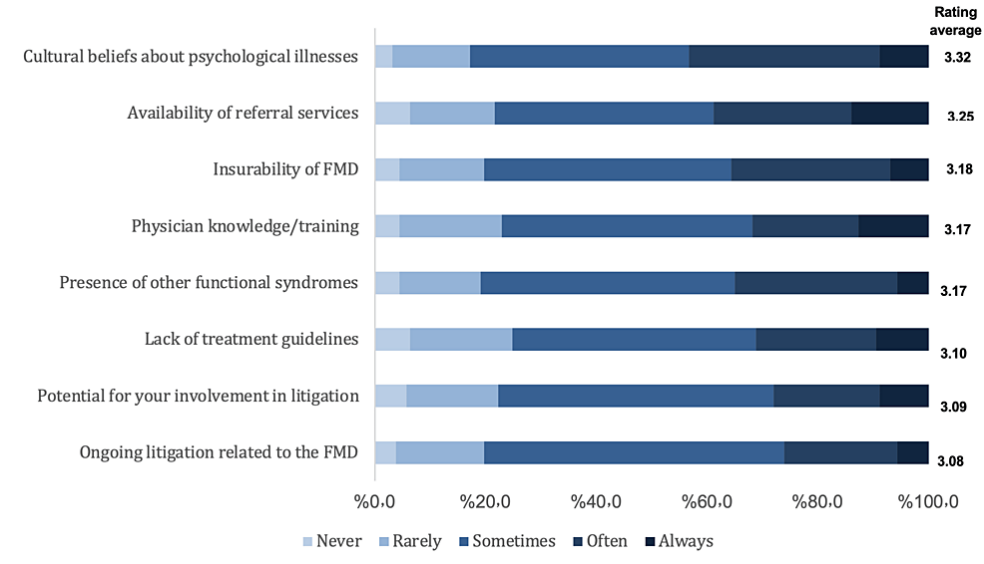
Restrictions in managing patients with FMD FMD: Functional movement disorders

**Figure 4:**
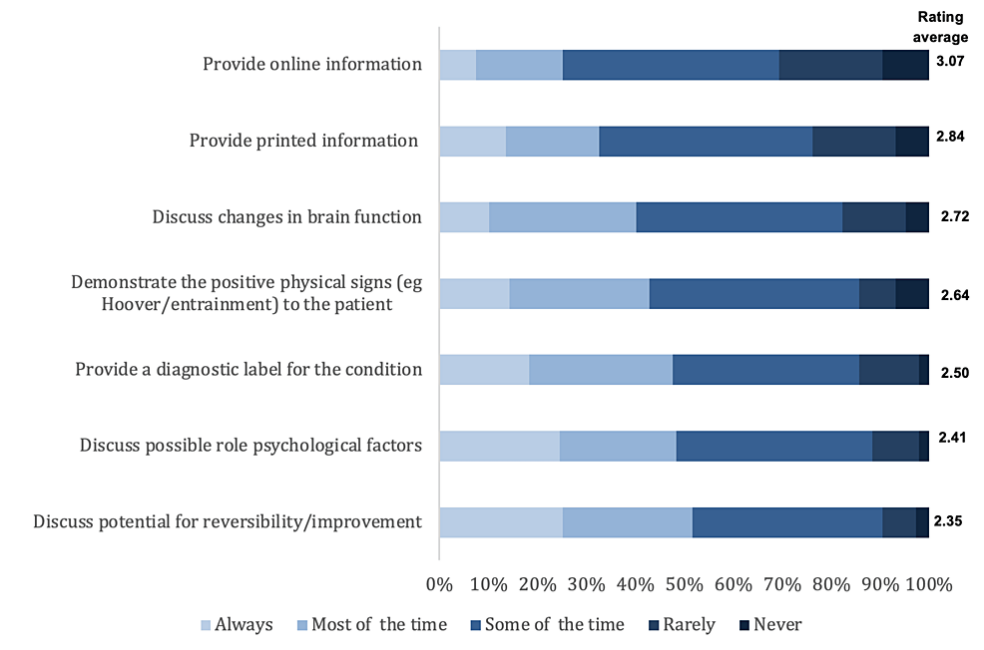
Education methods of the patient about FMD diagnosis FMD: Functional movement disorders

We present here the results of the opinions and clinical practice about diagnosing and managing FMD. As for the influence of predictors for non-FMD diagnosis, the highest rating was for evidence of physical injury (3.15 out of 5) and the lowest rating was for the male gender (2.69 out of 5). Figure 1 illustrates the influence of predictors for the non-FMD diagnosis. As for the effectiveness of treatment strategies of FMD, the highest rating was for educating the patient (3.46 out of 5) and the lowest was for alternative or complementary medicine (2.66 out of 5). The effectiveness of treatment strategies for FMD is shown in Figure 2. As for restrictions in managing patients with FMD, the highest rate was for cultural beliefs about psychological illnesses (3.32) and the lowest rate was for ongoing litigation related to the FMD (3.08). Figure 3 illustrates the restrictions in managing patients with FMD. Lastly, regarding educating the patient about FMD diagnosis, the highest rating was for providing online information (3.07), and the lowest rating was for discussing the potential for reversibility/improvement (2.35). Figure 4 shows the education methods of the patient about FMD diagnosis.

**Figure 5:**
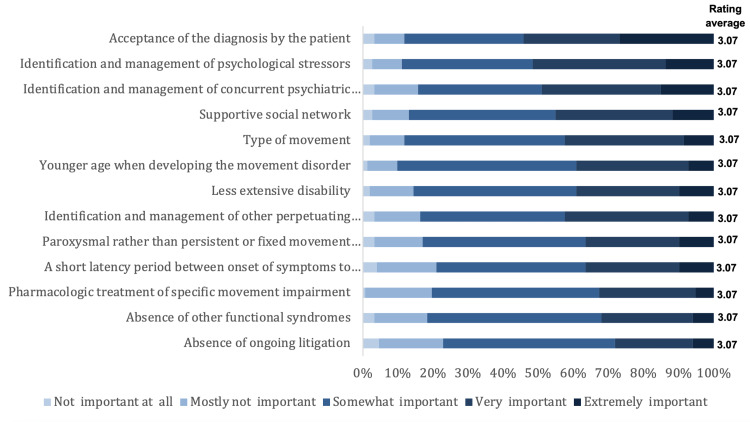
Importance of predictors for a better prognosis of FMD Choices were listed in descending order by average ratings. FMD: Functional movement disorders

Figure 5 illustrates the importance of predictors for a better prognosis of FMD. Choices were listed in descending order by average ratings. Acceptance of the diagnosis by the patient, identification and management of psychological stressors, identification and management of the concurrent psychiatric disorder, supportive social network, type of movement, younger age when developing movement disorder, and less extensive disability played important roles in a better prognosis. In the contrast, pharmacologic treatment of specific movement impairment, absence of other functional syndromes, and absence of ongoing litigation might be minor predictors.

**Figure 6:**
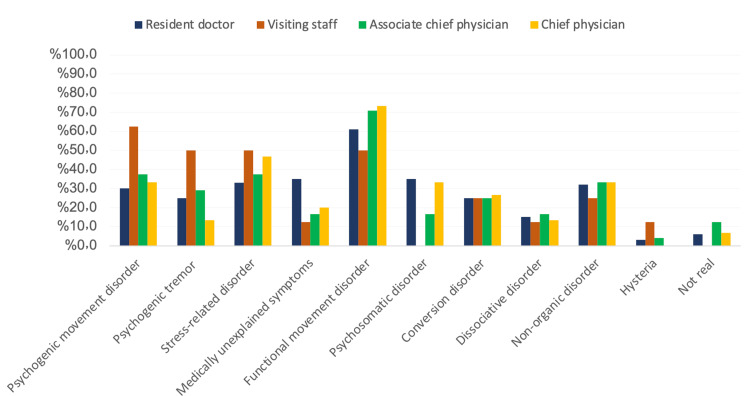
The preferred terms in communicating with medical professionals according to the title

Figure 6 shows the preferred terms for communicating with medical professionals according to the title. Among residents, the most used term was FMD. The most used among visiting staff was PMD. Among associate physicians was FMD, and among chief physicians was FMD. Generally, the most used term in this study was FMD.

## Discussion

The study aimed to assess the knowledge and clinical practices of FMDs among neurologists in Saudi Arabia. It is evident that neurologists in the country hold different opinions, with a variety of clinical practices being used. This can be partly explained by the cultural beliefs about psychological illness and the unavailability of referral services.

There is a high number of physicians in our sample treating FMDs without prior fellowship training in movement disorders, which may explain the lack of following established guidelines in approaching FMDs. It appears that the number of years in fellowship training, and years of practice in movement disorders affect the number of patients assessed per month. Clinicians with six or more years of practice in movement disorders see more patients than those with less than six years.

The term “functional movement disorder” was used by most respondents of this study. Even though they use the same terminology, the opinions and management have differed. Many treatment strategies are being practiced with variable effectiveness which is consistent with a recent Chinese study [9]. This could also be due to the scarcity of guidelines. In contrast to the Chinese study, this study shows higher effectiveness of treatment through patient education via providing online information over psychotherapy with antidepressants or anxiolytic treatment [9]. Acceptance of the diagnosis by the patient and diagnosis at an early age are two of the key predictors of a better prognosis, which is consistent with the Chinese study [9].

Regarding the frequency of patients with FMD encountered per month, 85 (36.8%) reported a rate of less than one patient per month, 59 (25.5%) reported one to three patients, 37 (16.0%) see four to six patients, and 12.6% were uncertain. These findings are almost compatible with the findings from a similar study, in which the majority of participants said they see no more than three FMD patients each month, whereas only a small proportion (18.5%) stated they see more than three FMD patients per month. Meanwhile, a percentage of (17.7%) were uncertain regarding the number of FMD patients they evaluated [11].

Predictors for non-FMD diagnosis

Considering the predictors for non-FMD diagnosis, the highest influential predictor was having evidence of physical injury and lack of psychiatric history of psychological stressors, while the lowest rating predictor was for the male gender. These findings are similar to another study with slight variabilities, in which more than half of the respondents considered that a previous diagnosis of an organic disorder provided by a trustworthy neurologist (59.9%), absence of related non-physiologic impairments (51.8%), and evidence of physical damage (50.0%) were influential factors for predicting a diagnosis other than FMD. Moreover, gender did not play a major role in predicting the diagnosis of FMD [12].

Management and treatment strategies

Regarding the efficacy of treatment strategies for FMD, the highest reported effective treatment strategy was patient education, while the least reported effective treatment strategy was integrative or complementary medicine. As for restrictions in managing patients with FMD, the highest rate was for cultural beliefs about psychological illnesses (3.32). Many clinicians in our study reported that rehabilitation services such as physiotherapy and occupational therapy are considered effective treatment strategies for FMD. Other prior investigations provided similar potentially efficient treatment approaches. For example, physiotherapy may contribute to the alleviation of symptoms. This was supported by a consensus on the significance of physiotherapy and occupational therapy in the multidisciplinary treatment of patients with FMD [13]. Moreover, this was similar to other investigations [14,15].

Predictors for a better prognosis of FMD

In our study, we found that the main predictors for a better prognosis of FMD are acceptance of the diagnosis by the patient followed by identification and management of psychological stressors, social support, the type of movement disorder, young age when developing the movement disorder, and less severe disability. Similarities to these findings were witnessed in previous studies. For instance, a study found that early FMD diagnosis, recognition and treatment of the coexisting psychiatric disease, and patient acceptance of the diagnosis all contributed significantly to a better prognosis. In contrast, social support and paroxysmal type might be minor predictors [8]. It was supported by another study, in which a main factor in predicting a better prognosis was the patient accepting their FMD diagnosis [16]. Also, the early diagnosis was considered by many professionals as a predictor of a better prognosis, which is corroborated by additional evidence [17].

Opinions on terminology

As for terminology, we found that among residents, associate chief physicians, and chief physicians, the most used term was FMD. However, the most used among visiting staff was psychogenic movement disorder. In a previous study, it was noted that the most common terminology used was "psychogenic movement disorder (PMD)". Additionally, "functional movement disorder" and "functional somatic syndrome" were also recognized by professionals. Other uncommon terms were hysterical disorders, stress-related disorders, and dissociative disorders [16].

This study tackled a critical issue with limited evidence-based data. We consider this study a valuable base for evidence as it is the first to be conducted in the region of Saudi Arabia and the Middle east. However, the study was limited by being a questionnaire, which inherently has the risk of recall, interviewer, and response bias. Also, as more neurologists with less training in the MDs specialization were enrolled for this study, sampling bias may be a limitation.

It is recommended to set up authentic guidelines for the diagnosis of FMD and ensure physician exposure to the approach to FMDs during and after training. It is also necessary to decide on universally accepted terminology, which would be in line with international standards.

## Conclusions

Our study concluded that evidence of physical injury and lack of psychiatric history of psychological stressors are considered predictors for a diagnosis other than FMD. The main reported effective treatment strategies for FMD include patient education and rehabilitation services. Additionally, acceptance of the diagnosis by the patient, identification and management of psychological stressors and concurrent psychiatric disorder, and social support are important factors for a better prognosis. FMDs and PMDs were the used terminologies. Combined efforts are needed to promote more regional research on this topic.
